# Supplementary material for: Variant U1 snRNAs contribute to cell cycle and differentiation control of human iPS cells
Source: Nat Commun. 2026 May 13;17:4334. doi: 10.1038/s41467-026-73121-0 (PMC13172311; doi:10.1038/s41467-026-73121-0)
Supplement: Supplementary file 1 — Supplementary Information [file 41467_2026_73121_MOESM1_ESM.pdf]

SUPPLEMENTARY INFORMATION

Variant U1 snRNAs contribute to cell cycle and differentiation control of human iPS cells

Yajie Zhu[1,#], Konstantinos Sofiadis[1,2,4,#], Athanasia Mizi[1], Vasilisa Kalinkina[1], Matthias Akyel[1], Milos Nikolic[2,5], Lukas Cyganek[3], Carmelo Ferrai[1], Argyris Papantonis[1,2,*]

[1] Institute of Pathology, University Medical Center Göttingen, Göttingen, Germany
[2] Center for Molecular Medicine Cologne, University of Cologne, Cologne, Germany
[3] Stem Cell Unit, Clinic for Cardiology and Pneumology, University Medical Center Göttingen, Göttingen, Germany
[4] Princess Maxima Center for Pediatric Oncology, Utrecht, The Netherlands
[5] DISCO Pharmaceuticals, Cologne, Germany
[#]These authors contributed equally to this work

*Correspondence to: A.P.; e-mail: argyris.papantonis@med.uni-goettingen.de

- This file contains **Supplementary Figs 1-5** and **Supplementary Tables 1-3**.

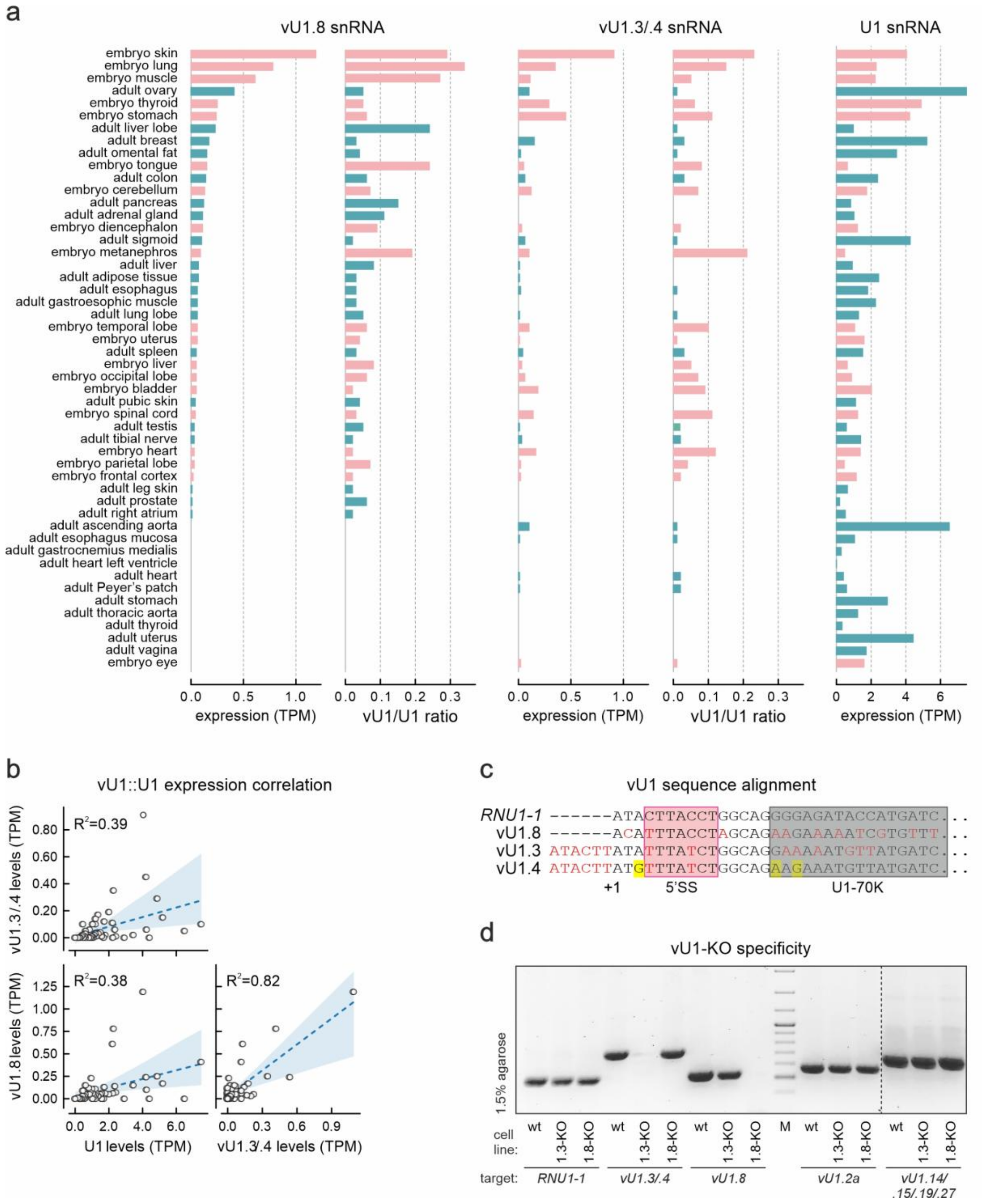

Supplementary Fig 1. Expression of vU1 snRNAs across human cell types. a, Bar plots showing the mean expression levels (from at least three biological replicates per cell type) of vU1.3/.4, vU1.8, and canonical U1 snRNA in embryonic (pink) and adult human tissues (green) from ENCODE total RNA-seq data[53]. **b**, Plots showing correlations of variant and canonical U1 snRNA levels from the data in panel a. **c**, Alignment of the vU1.3/.4, vU1.8, and canonical U1 sequences around the region that interacts with donor splice sites on pre-mRNAs (pink). Sequence deviations between vU1.4 and vU1.3 are highlighted (yellow). **d**, Electrophoresis of gDNA PCR products showing specificity of CRISPR-mediated vU1-KOs; this experiment has been repeated twice. M: Size marker profile.

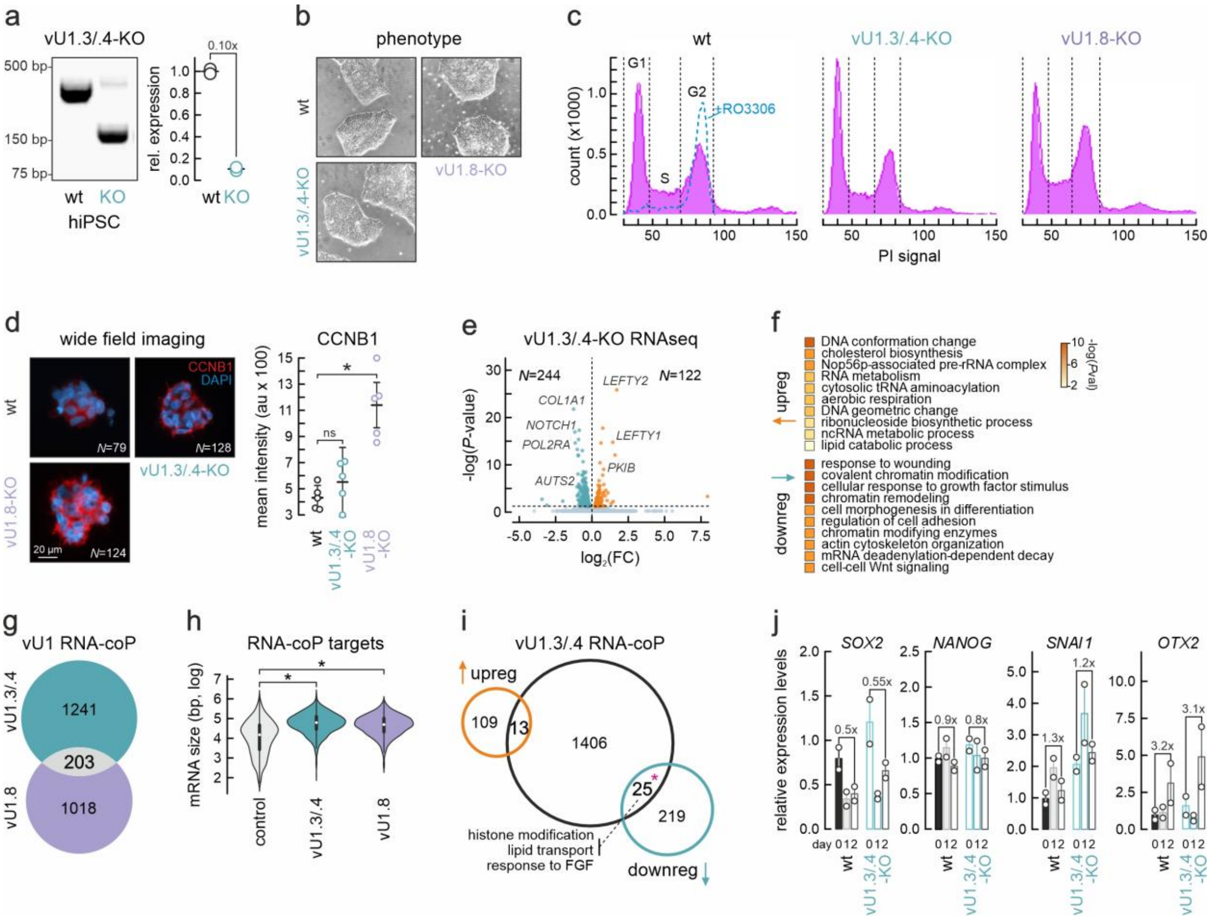

Supplementary Fig 2. Effects of the vU1.3/.4 knockout in hiPSCs. a, Left: Electrophoretic profiles of PCR products corresponding to the wild-type (wt) and near-complete knocked-out vU1.3/.4 loci. Size marker positions are indicated. Right: RT-qPCR data from two independent replicates showing changes (normalized mean±SD) in vU1.3/.4 expression in KO relative to wt hiPSCs. **b**, Representative bright field images (from at least three independent experiments) of wild-type, vU1.3/.4- and vU1.8-KO hiPSC colonies. **c**, Cell cycle profiles of PI-stained wild-type, vU1.3/.4- and vU1.8-KO hiPSCs. Treatment with a CDK1 inhibitor (RO3306; blue dashed line) provide a control of G2/M-arrested cells. **d**, Left: Representative images of asynchronous wild-type, vU1.3/.4- and vU1.8-KO hiPSCs immunostained for CCNB1 and counterstained by DAPI from two independent experiments. Right: Quantification of CNNB1 levels (mean±SD) per area of all cells (*N*) in 5 independent frames. *$P<0.01$, unpaired two-tailed Student's t-test. **e**, Volcano plot showing differentially up- (orange) and downregulated genes (green) in vU1.3/.4-KO hiPSCs given a cutoff of $P_{adj}<0.05$. **f**, Plot showing the top GO terms associated with the vU1.3/.4-KO DEGs from panel e. **g**, Venn diagram showing the overlap of transcripts co-purified with vU1.3/.4 (green) and vU1.8 (purple) in RNA-IP experiments performed in triplicates and merged. **h**, Violin plots (center shows the median, rectangles indicate the 25th and 75th percentile values, and the plots extend to 1.5x this interquantile range) showing the distribution of lengths for transcripts co-purified with vU1.3/.4 (green) and vU1.8 (purple) compared to control IPs. **i**, Venn diagram showing the overlap of differentially expressed genes from panel c with transcripts co-purified with vU1.3/.4. *$P<0.05$, one-tailed hypergeometric test. **j**, Bar plots showing changes (relative to the mean of day 0 ±SD from two independent replicates) in expression levels of the indicated marker genes for control (wt) and vU1.3/.4-KO hiPSCs at 1 or 2 days of differentiation.

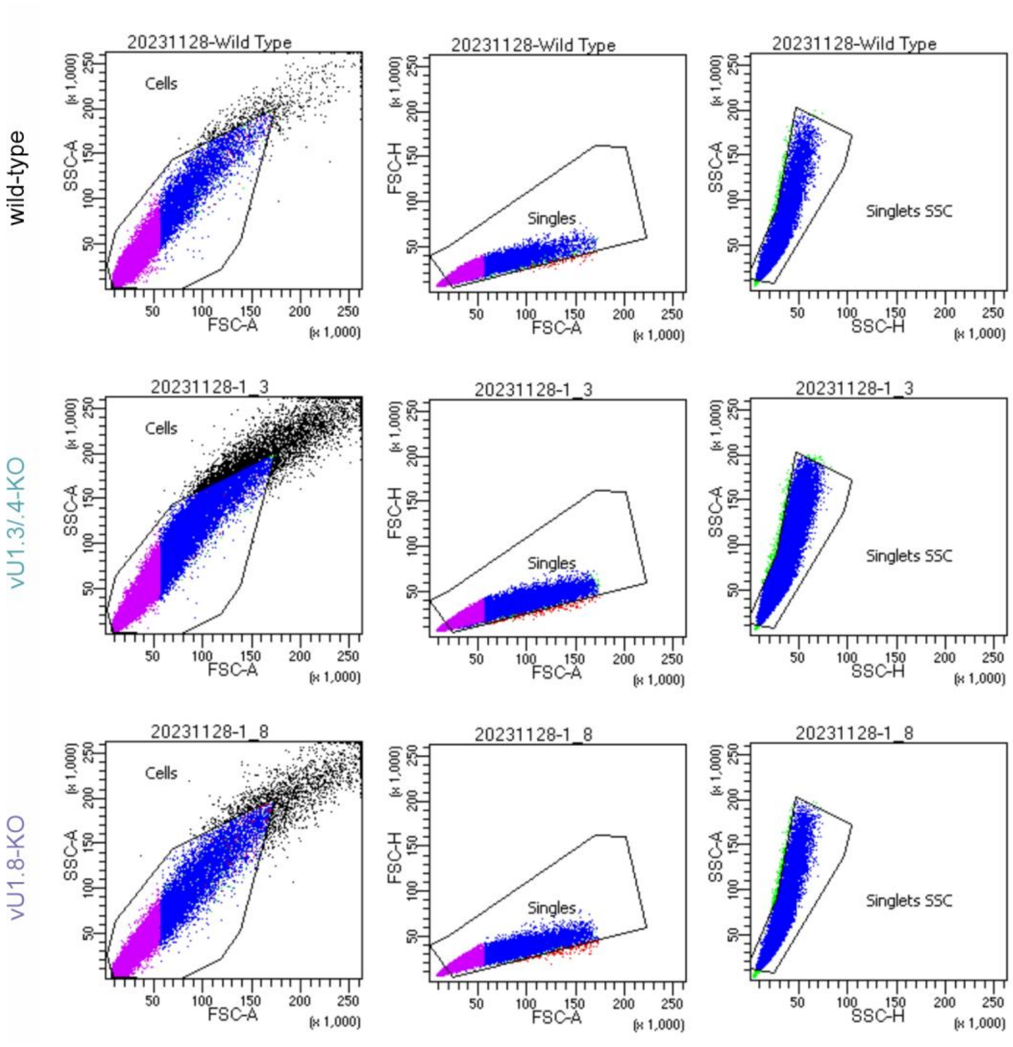

Supplementary Fig 3. Gating strategy for hiPS cell storing. Plots showing the gating used for sorting PI-stained wild-type (top), vU1.3/4- (middle) or vU1.8-KO hiPSCs and estimating cell cycle profiles in each population.

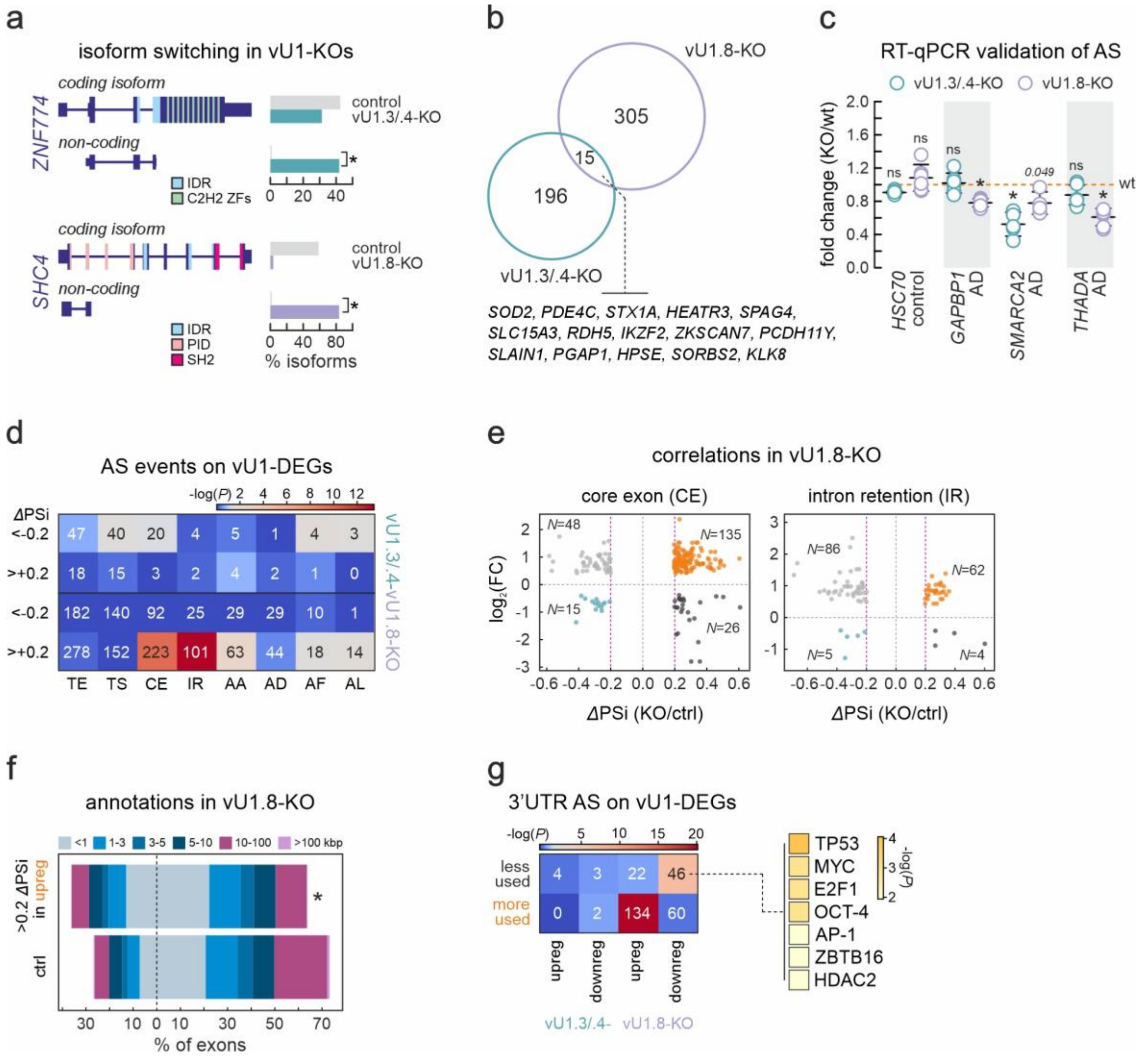

Supplementary Fig 4. Alternative splicing effects following vU1-KOs in hiPSCs. a, Changes in the levels of coding and non-coding isoforms of *ZNF774* and *SHC4* in control (grey), vU1.3/.4- (green) or vU1.8-KO cells (purple). *P<0.01, two-tailed Fisher's exact test. **b**, Venn diagram of the overlap between genes exhibiting isoform switching in each vU1-KO. The 15 genes shared between the two KO lines are listed (below). **c**, Plot showing RT-qPCR measurements (mean±SD from at least three independent replicates per condition) of AD usage relative to wild-type and normalized to *YWHAZ* (orange dotted line) upon vU1.3/.4- (green) or vU1.8-KO (purple) in five exemplary mRNAs. *HSC70* provides a negative AS control. * P<0.01, unpaired two-tailed Student's t-test. **d**, Heat map showing the number of events per AS type for differentially expressed genes (DEGs) in vU1.3/.4- (top) or vU1.8-KO hiPSCs (bottom). The color scale reflects statistical overrepresentation (-log$P$) in each case. **e**, Plot showing correlation between gene expression (log_2FC) and splicing changes (ΔPSi) of vU1.8-KO-associated transcripts displaying cassette exon (CE) or intron retention events (IR). **f**, Plot showing the percent of exons that showing differential usage in vU1.8-KO hiPSCs and are located at increasing distances from TSSs compared to non-AS exons. *P<0.01, two-tailed Wilcoxon-Mann-Whitney test. **g**, Left: As in panel d, but for alternative 3' end usage in each knockout line. Right: Enrichment for genes regulated by the indicated transcription factors (based on TTRUST[65]).

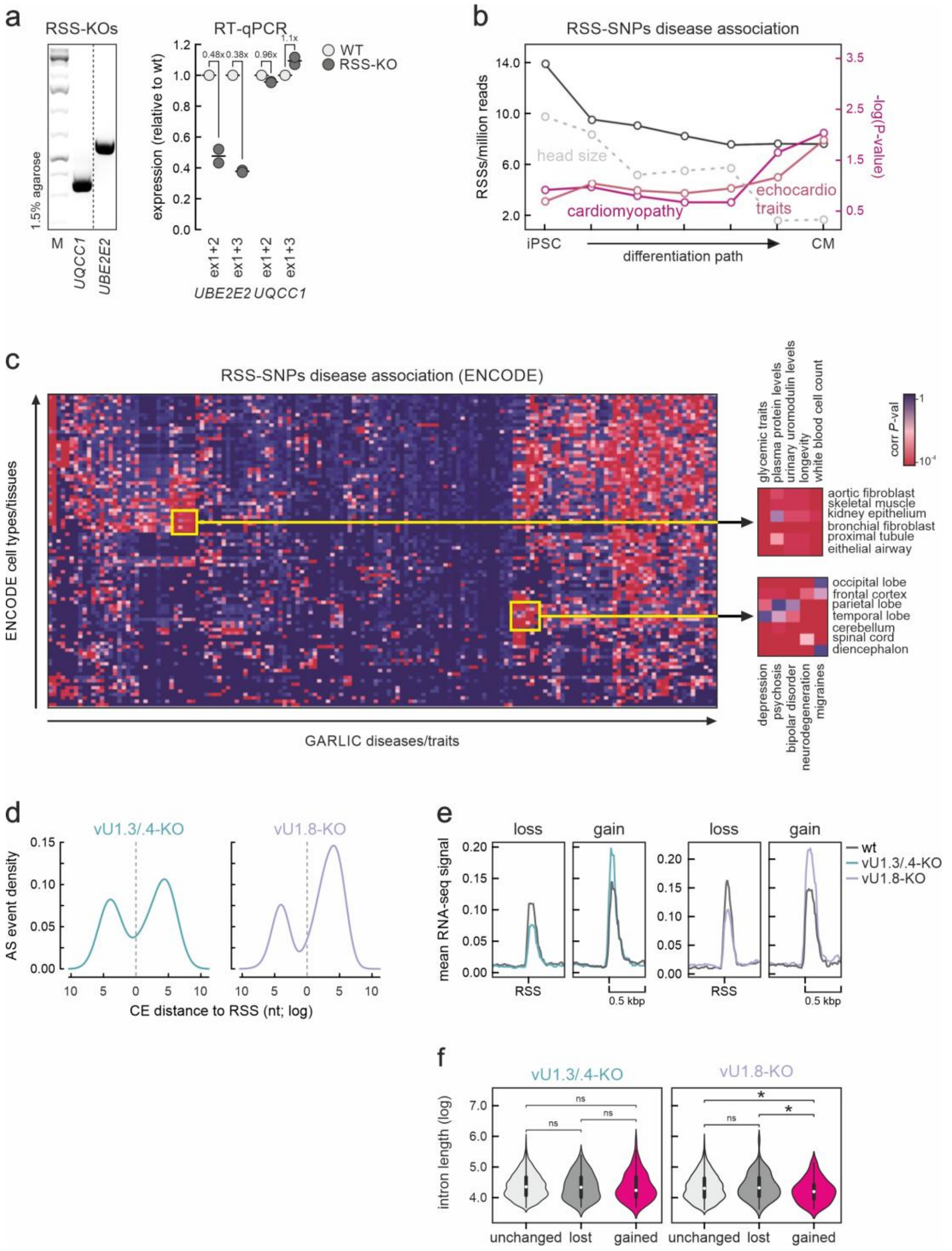

Supplementary Fig 5. Features of recursive splicing sites in human cells. a, Left: Electrophoresis of genomic PCR-validated RSS deletions in two different gene loci. M: Size marker profile. Right: Plots showing fold-changes in the mRNA levels (mean ±SD from two independent replicates) of two exemplary genes upon RSS-KO using primers in the indicated exons. **b**, Plot showing the number of RSSs discovered per million RNA-seq reads (black line) along the differentiation of hiPSCs into cardiomyocytes (RNA-seq data from ref. [55]), and the enrichment for SNPs associated with cardiac disease (-log*P*; magenta/pink lines). Enrichments for SNPs associated with head size (dotted line) provide a negative control. **c**, Heatmap showing k-means clustered statistical association between RSSs in ENCODE cell/tissue types and SNPs linked to diseases and traits in the GARLIC database[54]. **d**, Plots showing the density of AS cassette exons up- and downstream of RSSs in vU1.3/.4- (left) and vU1.8-KO hiPSCs

(right). **e**, Line plots showing mean RNA-seq coverage in the 1 kbp around RS-exons lost or gained upon vU1.3/.4- (left) or vU1.8-knockout (right). **f**, Violin plots (center shows the median, rectangles indicate the 25th and 75th percentile values, and the plots extend to 1.5x this interquantile range) of the lengths of introns carrying RSSs that do not change (light grey), are lost (dark grey) or gained (magenta) upon each vU1-KO hiPSC line.

Supplementary References

65. Han, H., et al. TRRUST v2: an expanded reference database of human and mouse transcriptional regulatory interactions. *Nucleic Acids Res.* **46**, D380–D386 (2018).

Supplementary Table 1. gRNAs used for the knockout of genomic loci.

Target locus	gRNA 1	gRNA 2
RNU1-3/-4	GTGTTAATACCGCTCGAGCTC	CGAGCTCGAGCGGTATTAACA
RNU1-8	ACTTGCCCCGCGCACCCTCGAGG	CAGTCTATCGTGTTATATCCTGG
UBE2E2	AGATGGGGTTTCGGTCGCCC	AAGTATGTAAAGGAATATTG
UQCC1	GAGGGCTGACTCTTCAATTC	ATTCTATTTTTTCTTCGTTA

Supplementary Table 2. Primers used for validation PCRs on genomic DNA.

Target gene	Forward primer	Reverse primer
vU1.3/.4	GCTGCTTTGCCACGAAAG	CAGTAAGCCATGCACTCC
vU1.8	GACATCAGGGATAGGGCGAC	CGGCAAGGACAACAACCAAC
RNU1-1	TTCGCCACGAAGGGAGTTC	ATACGGCTGATGCTCGCTTT
vU1.2a	TCACGGATAGGGCGACTTCT	TAAACCGGCTGACACTCCTT
vU1.14/.15/.19/.27	CGTTGCGAACGGAAACAACT	CACACCTCCCCAAAGGACAA
UBE2E2	AGATGGTGAGGAGGGGACAA	ATGCCCAGGTACAATGGCTC
UQCC1	AGGTAAAACATGACTGCGTAGACT	ACAGTAGATGGTAGGACAGCC

Supplementary Table 3. Primers used in RT-qPCR.

Target gene	Forward primer	Reverse primer
EOMES	CGGCCTCTGTGGCTCAAA	AAACATGCGCCTGCCCT
GAPDH	CACCCACTCCTCCACCTTTGAC	TCCACCACCCTGTTGCTGTAG
KLF4	ACCAGGCACTACCGTAAACACA	GGTCCGACCTGGAAAATGCT
NANOG	ACAACTGGCCGAAGAATAGCA	GGTTCCCAGTCGGGTTCAC
OTX2	CGCCACATCTACTTTGATAGCTG	GGTTCAGAGTCCTTGGTGGG
SNAI1	GGCCTAGCGAGTGGTTCTTC	CTGCTGGAAGGTAAACTCTGGAT
SOX2	TGCGAGCGCTGCACAT	GCAGCGTGTACTTATCCTTCTTCA
TBXT	TATGAGCCTCGAATCCACATAGT	CCTCGTTCTGATAAGCAGTCAC
YWHAZ (ex3-4)	GCTTCACAAGCAGAGAGCAAAGT	GGTATGCTTGTTGTGACTGATCG
HSC70 (ex1-2)	TTATTGGAGCCAGGCCTACAC	GCGACATAGCTTGGAGTGGT
GAPBP1 (ex5-6)	CAGAAAATTCATCCAAGGCAACAG	TGTGGCCACTACTGGAGTTTC
SMARCA2 (ex2)	GATGGGGAGGCTGATTGGTAG	AAGAATTGGCCCAGGGGAAG
THADA (ex1-2)	TGAAGCAGCGTTCTAAAGGG	AAGGTCCTGATGGCAAATGG
TOP2A (3'UTR)	TGCTCAGCAATGAGCTATTAGA	ACATTACTCAAGTCACACACA
SPCS2 (ex3-4)	TCGCCTCACCATCTGTACAATC	TGGTCAGAATCCCCATCATCAC
