# Supplementary material for: Variant U1 snRNAs contribute to cell cycle and differentiation control of human iPS cells
Source: Nat Commun. 2026 May 13;17:4334. doi: 10.1038/s41467-026-73121-0 (PMC13172311; doi:10.1038/s41467-026-73121-0)
Supplement: Supplementary file 2 — Description of Additional Supplementary Files [file 41467_2026_73121_MOESM2_ESM.pdf]

Description of Additional Supplementary Files

Supplementary Data 1. Lists of differentially expressed genes in vU1.3/.4- or vU1.8-KO hiPSCs.

Supplementary Data 2. List of RNAs copurifying with vU1.3/.4 or vU1.8 in hiPSCs

Supplementary Data 3. Output of the IsoformSwitch analysis in vU1.3/.4- or vU1.8-KO hiPSCs.

Supplementary Data 4. Output of the Whippet AS analysis in vU1.3/.4- or vU1.8-KO hiPSCs
