# Supplementary material for: Variant U1 snRNAs contribute to cell cycle and differentiation control of human iPS cells
Source: Nat Commun. 2026 May 13;17:4334. doi: 10.1038/s41467-026-73121-0 (PMC13172311; doi:10.1038/s41467-026-73121-0)
Supplement: Supplementary file 7 — Reporting Summary [file 41467_2026_73121_MOESM7_ESM.pdf]

nature portfolio

Corresponding author(s): PAPANTONIS, Argyris

Last updated by author(s): Apr 13, 2026

Reporting Summary

Nature Portfolio wishes to improve the reproducibility of the work that we publish. This form provides structure for consistency and transparency in reporting. For further information on Nature Portfolio policies, see our Editorial Policies and the Editorial Policy Checklist.

Statistics

For all statistical analyses, confirm that the following items are present in the figure legend, table legend, main text, or Methods section.

n/a	Confirmed	
☐	☒	The exact sample size (*n*) for each experimental group/condition, given as a discrete number and unit of measurement
☐	☒	A statement on whether measurements were taken from distinct samples or whether the same sample was measured repeatedly
☐	☒	The statistical test(s) used AND whether they are one- or two-sided<br>*Only common tests should be described solely by name; describe more complex techniques in the Methods section.*
☒	☐	A description of all covariates tested
☒	☐	A description of any assumptions or corrections, such as tests of normality and adjustment for multiple comparisons
☐	☒	A full description of the statistical parameters including central tendency (e.g. means) or other basic estimates (e.g. regression coefficient) AND variation (e.g. standard deviation) or associated estimates of uncertainty (e.g. confidence intervals)
☐	☒	For null hypothesis testing, the test statistic (e.g. *F*, *t*, *r*) with confidence intervals, effect sizes, degrees of freedom and *P* value noted<br>*Give P values as exact values whenever suitable.*
☒	☐	For Bayesian analysis, information on the choice of priors and Markov chain Monte Carlo settings
☒	☐	For hierarchical and complex designs, identification of the appropriate level for tests and full reporting of outcomes
☐	☒	Estimates of effect sizes (e.g. Cohen's *d*, Pearson's *r*), indicating how they were calculated

Our web collection on statistics for biologists contains articles on many of the points above.

Software and code

Policy information about availability of computer code

Data collection	Not applicable.
Data analysis	RNASeq3USP (https://github.com/christear/RNASeq3USP)<br>Gene Set Enrichment Analysis (GSEA; https://www.gsea-msigdb.org/)<br>STAR v.2.7.3a<br>htseq-count2 v.0.12.4<br>RUVseq package (doi:10.1038/nbt.2931)<br>DEseq2 v.1.34.0<br>Whippet (doi:10.1016/j.molcel.2018.08.018)<br>IsoformSwitchAnalyzeR (doi:10.1093/bioinformatics/btz247)<br>Metascape v.39 (doi:10.1038/s41467-019-09234-6)<br>NoiseSeq (doi:10.1093/nar/gkv711)<br>TTRUST (doi:10.1093/nar/gkx1013)<br>Recursive splicing detection (doi:10.1371/journal.pgen.1007579)<br>GARLiC association webtool (doi:10.1093/hmg/ddw423)<br>All statistical tests (Wilcoxon, hypergeometric) were performed in R, except Student's t-tests and Fisher's exact tests that were performed via the GraphPad (https://www.graphpad.com/quickcalcs/contingency1/).

For manuscripts utilizing custom algorithms or software that are central to the research but not yet described in published literature, software must be made available to editors and reviewers. We strongly encourage code deposition in a community repository (e.g. GitHub). See the Nature Portfolio guidelines for submitting code & software for further information.

Data

Policy information about availability of data

All manuscripts must include a data availability statement. This statement should provide the following information, where applicable:

- Accession codes, unique identifiers, or web links for publicly available datasets
- A description of any restrictions on data availability
- For clinical datasets or third party data, please ensure that the statement adheres to our policy

The RNA-seq data generated in this study have been deposited in the NCBI Gene Expression Omnibus repository under accession number GSE305587 [https://www.ncbi.nlm.nih.gov/geo/query/acc.cgi?acc=GSE305587].

Research involving human participants, their data, or biological material

Policy information about studies with human participants or human data. See also policy information about sex, gender (identity/presentation), and sexual orientation and race, ethnicity and racism.

Reporting on sex and gender	We have genome-edited and studied only a single hiPSC line originating from a female donor; although there is not reason to expect sex-specific contribution of vU1 regulation of splicing patterns, we cannot formally rule this possibility out.
Reporting on race, ethnicity, or other socially relevant groupings	Not applicable.
Population characteristics	Not applicable.
Recruitment	Not applicable.
Ethics oversight	Not applicable.

Note that full information on the approval of the study protocol must also be provided in the manuscript.

Field-specific reporting

Please select the one below that is the best fit for your research. If you are not sure, read the appropriate sections before making your selection.

[x] Life sciences [ ] Behavioural & social sciences [ ] Ecological, evolutionary & environmental sciences

For a reference copy of the document with all sections, see nature.com/documents/nr-reporting-summary-flat.pdf

Life sciences study design

All studies must disclose on these points even when the disclosure is negative.

Sample size	For CRISPR/Cas9-genome-edited hiPSC lines, at least two independent KO clones were analysed; for genomics experiments, at least three independent replicates were sequenced, as per common practice in the field.
Data exclusions	No data was excluded, with the exception of qPCR technical replicates showing abnormal melting curves.
Replication	All experiments, unless otherwise stated, were performed in at least two independent replicates and replication was successful.
Randomization	Not applicable.
Blinding	Blinding was only done between data generation and data analysis (samples were name-coded).

Reporting for specific materials, systems and methods

We require information from authors about some types of materials, experimental systems and methods used in many studies. Here, indicate whether each material, system or method listed is relevant to your study. If you are not sure if a list item applies to your research, read the appropriate section before selecting a response.

Materials & experimental systems

n/a	Involved in the study
[ ]	[x] Antibodies
[ ]	[x] Eukaryotic cell lines
[x]	[ ] Palaeontology and archaeology
[x]	[ ] Animals and other organisms
[x]	[ ] Clinical data
[x]	[ ] Dual use research of concern
[x]	[ ] Plants

Methods

n/a	Involved in the study
[x]	[ ] ChIP-seq
[ ]	[x] Flow cytometry
[x]	[ ] MRI-based neuroimaging

Antibodies

Antibodies used	anti-CCNB1 (Cell Signaling Technology, 4135; dil. 1:400)
Validation	Validation of specificity is provided by the manufacturer (see https://www.citeab.com/antibodies/123938-4135-cyclin-b1-v152-mouse-monoclonal-antibody)

Eukaryotic cell lines

Policy information about cell lines and Sex and Gender in Research

Cell line source(s)	Human induced pluripotent stem cells (hiPSCs; GM24581*B from Coriell) were derived from the preprogramming of human GM02036 fibroblasts by overexpressing the four Yamanaka factors (i.e., hOCT3/4, hSOX2, hKLF4 and hL-MYC) via episomal vectors, and validated for genomic integrity and their pluripotent character.
Authentication	Our iPSC cell line is authenticated by the Coriell Institute.
Mycoplasma contamination	All cell line stocks at the Institute of Pathology are tested for mycoplasma contamination via commercial kits twice per year. No contamination has been detected in our stocks to date.
Commonly misidentified lines (See ICLAC register)	Not applicable.

Plants

Seed stocks	*Report on the source of all seed stocks or other plant material used. If applicable, state the seed stock centre and catalogue number. If plant specimens were collected from the field, describe the collection location, date and sampling procedures.*
Novel plant genotypes	*Describe the methods by which all novel plant genotypes were produced. This includes those generated by transgenic approaches, gene editing, chemical/radiation-based mutagenesis and hybridization. For transgenic lines, describe the transformation method, the number of independent lines analyzed and the generation upon which experiments were performed. For gene-edited lines, describe the editor used, the endogenous sequence targeted for editing, the targeting guide RNA sequence (if applicable) and how the editor was applied.*
Authentication	*Describe any authentication procedures for each seed stock used or novel genotype generated. Describe any experiments used to assess the effect of a mutation and, where applicable, how potential secondary effects (e.g. second site T-DNA insertions, mosiacism, off-target gene editing) were examined.*

Flow Cytometry

Plots

Confirm that:

- [x] The axis labels state the marker and fluorochrome used (e.g. CD4-FITC).
- [x] The axis scales are clearly visible. Include numbers along axes only for bottom left plot of group (a 'group' is an analysis of identical markers).
- [x] All plots are contour plots with outliers or pseudocolor plots.
- [x] A numerical value for number of cells or percentage (with statistics) is provided.

Methodology

Sample preparation	PI-stained wt or vU1-KO hiPSCs were sorted to profile cells in different cell cycle stages.
Instrument	LSR Fortessa X-20, BD
Software	Cell Analyzer via the BD FACSDiva™ software (BD Biosciences).

Cell population abundance	Not applicable as cells were not sorted separately to use in downstream applications, just profiled.
Gating strategy	Please see Supplementary Fig 3, based on SCC/PI signal.

☒ Tick this box to confirm that a figure exemplifying the gating strategy is provided in the Supplementary Information.
